# Supplementary material for: Targeting repair pathways with small molecules increases precise genome editing in pluripotent stem cells
Source: Nat Commun. 2018 Jun 4;9:2164. doi: 10.1038/s41467-018-04609-7 (PMC5986859; doi:10.1038/s41467-018-04609-7)
Supplement: Supplementary file 1 — Supplementary Information [file 41467_2018_4609_MOESM1_ESM.pdf]

SUPPLEMENTARY INFORMATION

Targeting repair pathways with small molecules increases precise genome editing in pluripotent stem cells

Riesenberg et al.

Supplementary Figures and Tables

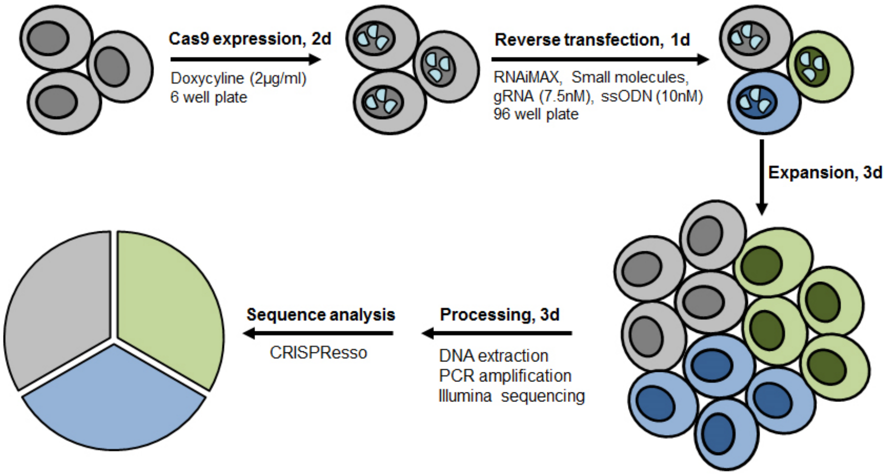

Supplementary Fig. 1: Genome editing and analysis flowchart. 409-B2 iCRISPR hiPSCs are treated with 2µg/ml doxycycline for 2 days to induce Cas9 or Cas9n expression. Reverse transfection with RNAiMAX, gRNA (7.5nM each), ssODN donor (10nM) and the small molecules to be evaluated is carried out in a 96 well plate for 1 day. Cells are then expanded for 3 days with regular media change. After harvest follows DNA extraction, PCR amplification of targeted loci, Illumina sequencing and CRISPResso[1] sequence analysis for amount of indels and targeted nucleotide substitutions (TNS).

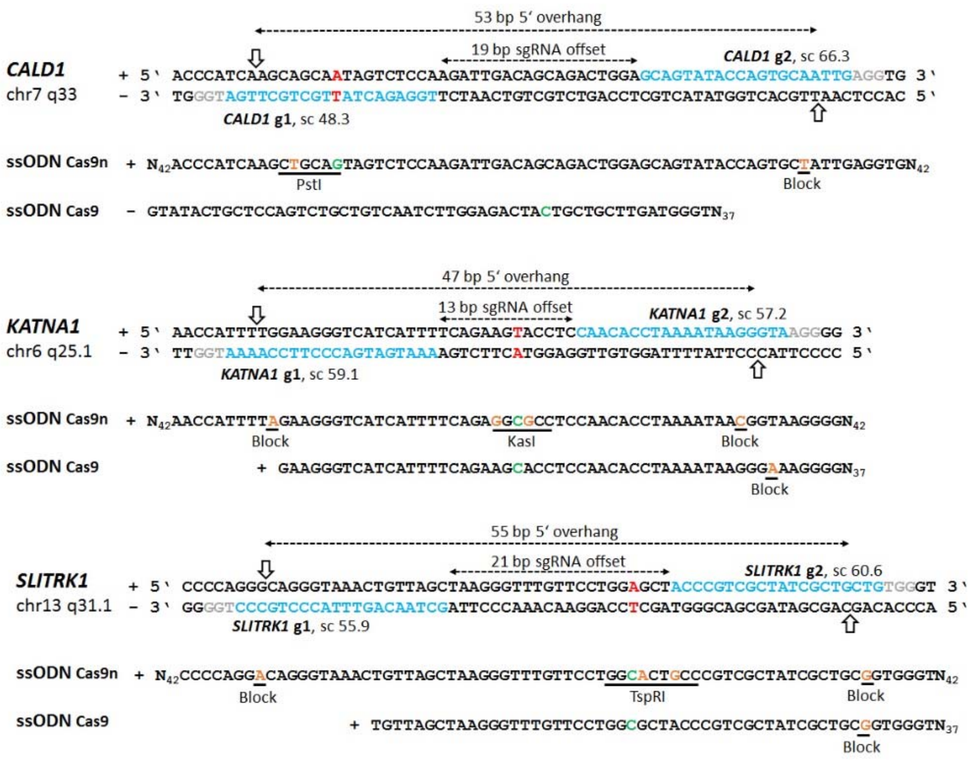

Supplementary Fig. 2: Design of gRNAs and ssODN for targeted nucleotide substitution (TNS) of human *CALD1*, *KATNA1* and *SLITRK1*. Shown are the respective loci of *CALD1*, *KATNA1* and *SLITRK1* together with the gRNAs and their efficiency score (sc) (sgRNA scorer 1.0[2]) used for DSB generation. The PAM site is grey, the target sequence is blue and the base to be changed is red. The point of nick by Cas9n or DSB by Cas9 is indicated by an arrow. Whereas both guides are used for editing with Cas9n, *CALD1* g1, *KATNA1* g2 and *SLITRK1* g2 are used for editing with Cas9. The respective ssODN for editing with both Cas9 variants are also shown. Desired mutation is marked green and additional mutations are orange. ‚Block' indicates a Cas9-blocking mutation to prevent re-cutting oft the locus. All Cas9n donors have 50nt homology arms after the nicks while all Cas9 donors are 90nt in total with the desired mutation centered in the middle. The full sequences are shown in Supplementary Table 3.

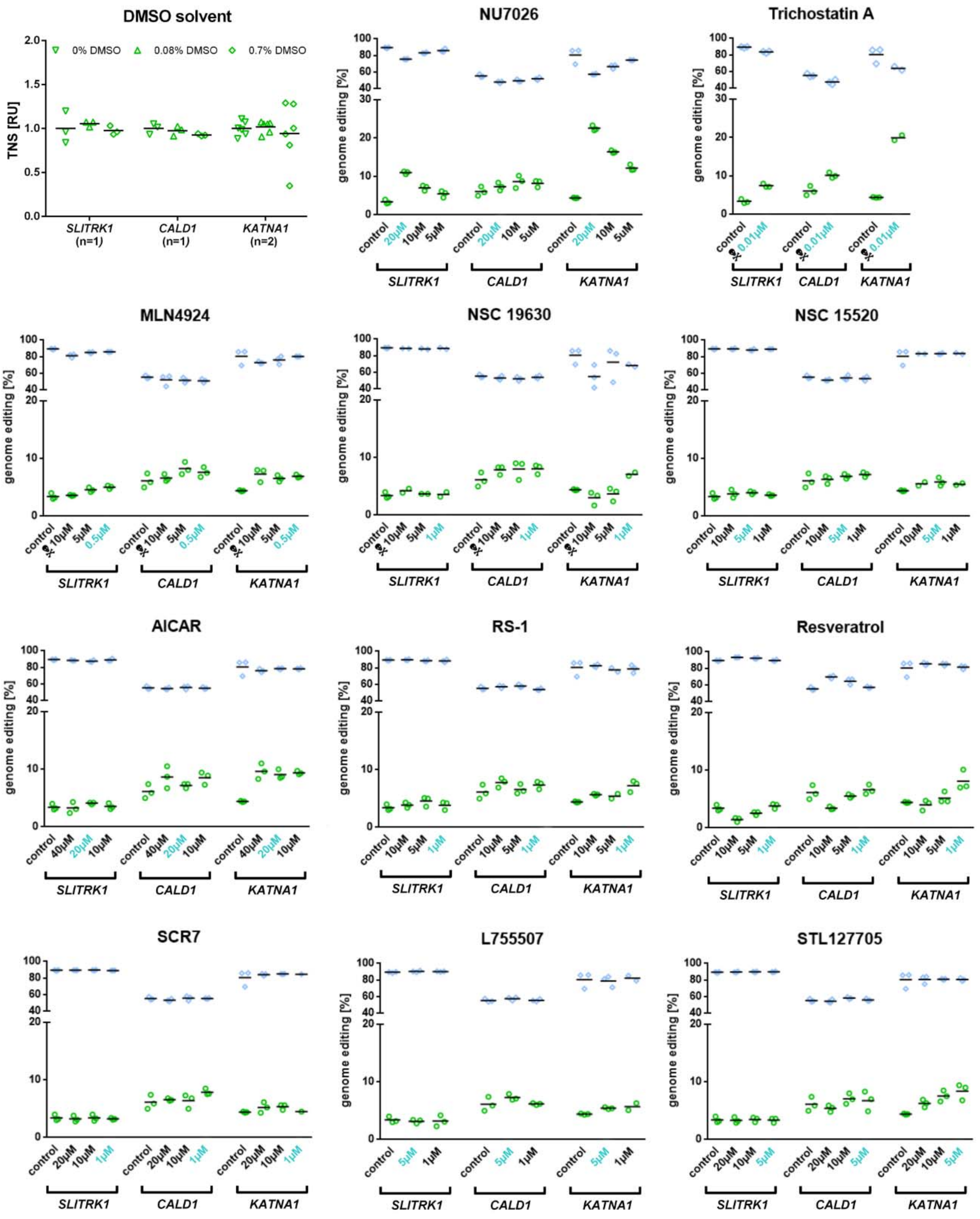

Supplementary Fig. 3: First screen for solvent effect and influence of different small molecule concentrations on genome editing in iCRISPR hiPSCs. Shown are genome editing efficiencies in *CALD1, KATNA1* and *SLITRK1* in 409-B2 iCRISPR-Cas9n hiPSCs. Targeted Nucleotide Substitutions (TNS) are green and indels are blue. Each symbol represents a technical replicate. The respective means are shown as a black line. Each skull indicates cell death of up to around 20% determined by phase contrast light microscopy. All cells died with 1µM and 0.1µM Trichostatin A. Concentrations chosen for further experiments are marked with turquoise.

A **Acute toxicity**

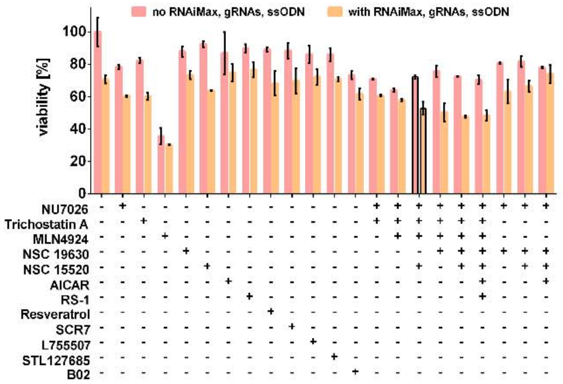

B **Long-term genotoxicity**

5x (1 day passaging with mock <u>or</u> CRISPY mix, and RNAiMax; 3 days recovery)

sample	metaphases	band number	gray shades	healthy karyotype (46,XX)
mock (bulk)	20	400	3	20/20
CRISPY (bulk)	25	450	3	25/25
mock clone 1	25	400	3	21/25 (4 polyploid)
mock clone 2	25	450	3	25/25
mock clone 3	25	450	3	25/25
mock clone 4	25	450	3	25/25
mock clone 5	25	400	3	25/25
CRISPY clone 1	25	400	3	25/25
CRISPY clone 2	25	400	3	25/25
CRISPY clone 3	25	400	3	25/25
CRISPY clone 4	25	400	3	22/25 (3 polyploid)
CRISPY clone 5	25	400	3	25/25

Supplementary Fig. 4: Toxicity of the CRISPY mix and its components. A resazurin assay from 409-B2-iCRISPR-Cas9n cells after 24h incubation with the small molecules and combinations from Fig. 2 and 3, with (orange) and without RNAiMax, gRNA and ssODNs (pink), is shown in (A). Resazurin is converted into fluorescent resorfin by cellular dehydrogenases and resulting fluorescence (Exitation: 530-570nm, Emission: 590-620nm) is considered as a marker for cell viability[3]. Resorfin absorption (610±30nm) of normally passaged cells without small molecule treatment and RNAiMax, gRNAs, and ssODN is set to 100% cell viability. The CRISPY mix is highlighted with black borders and is slightly toxic with no additive toxic effect of its components. Error bars show the standard deviation of two technical replicates. Karyotype analysis after five rounds of passaging the cells together with the CRISPY mix and mock treatment is shown in (B). At least 20 metaphases of the bulk and five clones of each conditions were analysed using trypsin-induced Giemsa staining. No numerical or large scale chromosomal aberrations, except for a small subset of metaphases from two single clones corresponding to CRISPY mix (3 of 25 metaphases polyploid) and mock treatment (4 of 25 metaphases polyploid), were identified.

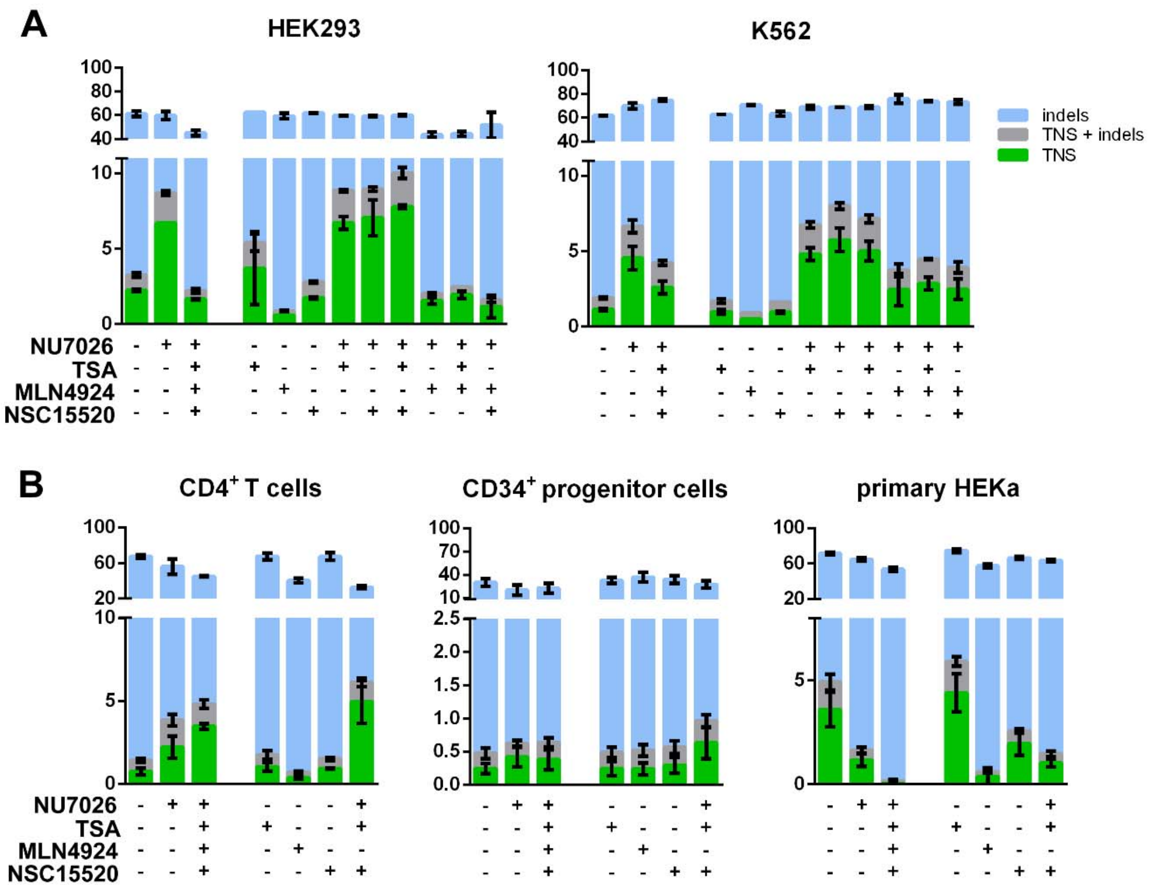

Supplementary Fig. 5: Impact of the CRISPY mix and small molecule combinations on Targeted Nucleotide Substitution (TNS) efficiency in non-pluripotent cell types. Shown are TNS efficiencies in *HPRT* with electroporated Cpf1 ribonucleoprotein and ssODN. All possible combinations for the CRISPY mix components are shown for HEK293 and K562 cells (A). While NU7026 increases TNS efficiency, TSA and NSC15520 have no clear effect, and MLN4924 has a clear disruptive effect in immortalized cell lines. MLN4924 has a disruptive effect on TNS efficiency in primary cells as well (B). The CRISPY mix without MLN4924 has a higher effect on TNS efficiency than NU7026 alone in $CD4^+$ T and $CD34^+$ progenitor cells. In primary Human Epidermal Keratinocytes (HEKa) also NU7026 and NSC15520 have a disruptive effect on TNS efficiency. Shown are TNS, TNS + indels, and indels with green, grey or blue bars, respectively. Error bars show the standard deviation of two independent experiments for HEK293 and K562 cells, three independent experiments for $CD4^+$ T cells, two technical replicates for each of four independent experiments for $CD34^+$ progenitor cells, and two technical replicates for each of two independent experiments for primary HEKa cells.

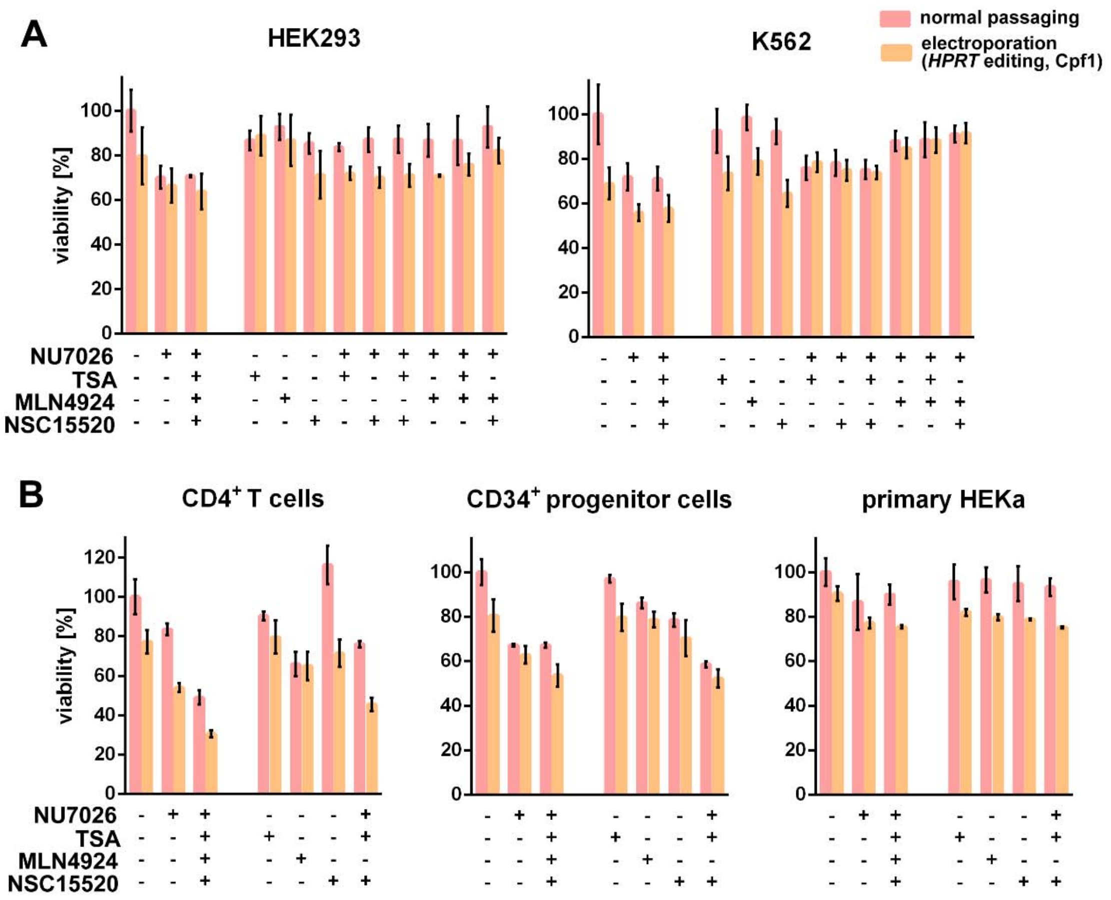

Supplementary Fig. 6: Impact of the CRISPY mix and small molecule combinations on cell viability in non-pluripotent cell types. Results of resazurin assays for cell viability after 24h incubation with the small molecules and combinations from Supplementary Fig. 5 are shown for HEK293, and K562 cells (A) and $CD4^{+}$ T, $CD34^{+}$ progenitor, and primary HEKa cells (B). Resazurin is converted into fluorescent resorfin by cellular dehydrogenases and resulting fluorescence (Exitation: 530-570nm, Emission: 590-620nm) is considered as a marker for cell viability[3]. Resorfin absorption (610±30nm) of normally passaged cells without small molecule treatment is set to 100% cell viability. Shown are viability after small molecule treatment following normal passaging (pink) and editing of *HPRT* using electroporated Cpf1 ribonucleoprotein and ssODN (orange), respectively. Error bars show the standard deviation of three independent experiments.

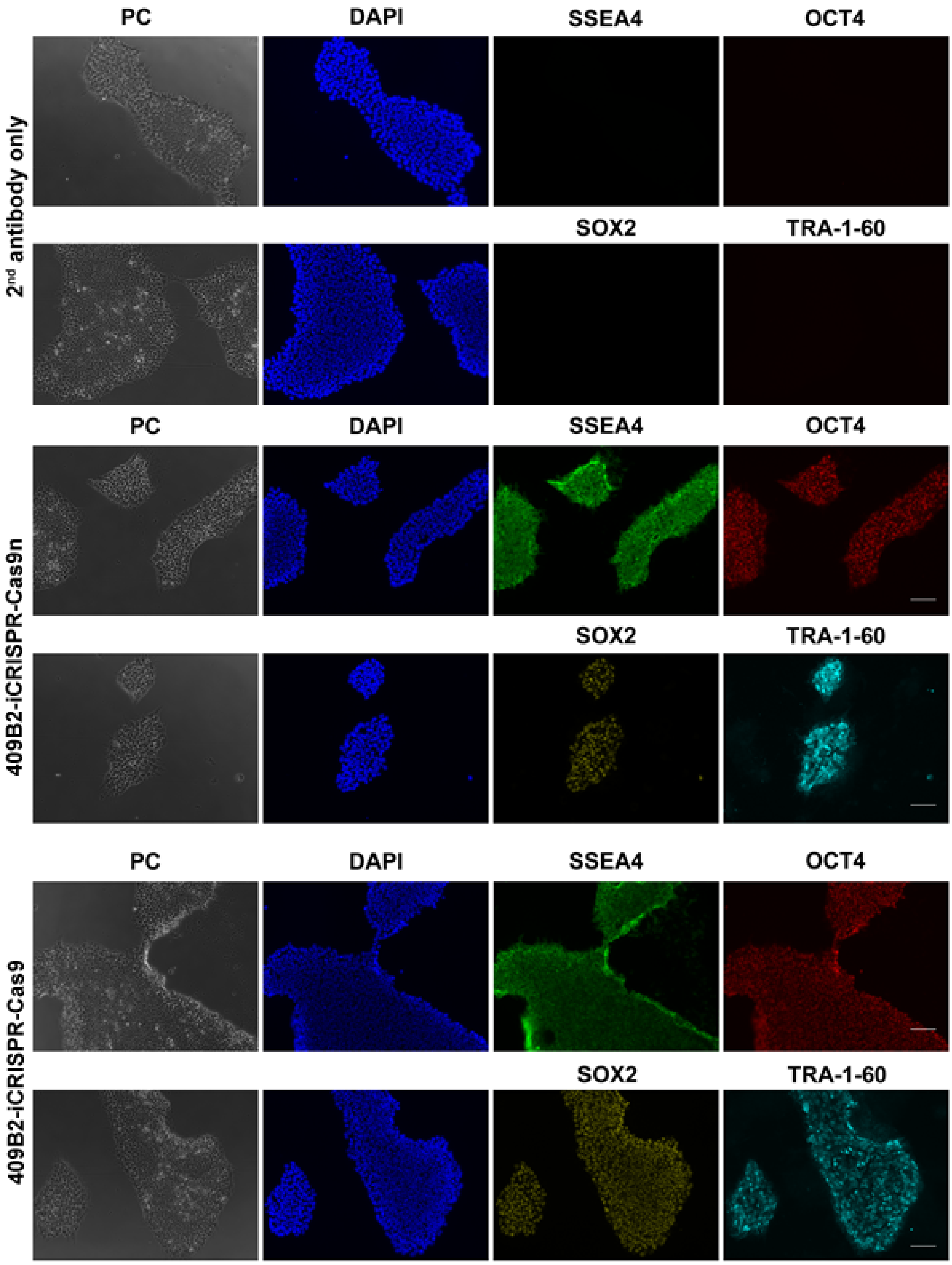

Supplementary Fig. 7: Expression of pluripotency markers in 409-B2 iCRISPR cell lines. Representative images of the phase contrast (PC), nuclei stain DAPI (blue), and pluripotency markers SSEA4 (green), OCT4 (red), SOX2 (yellow) and TRA-1-60 (turquoise) are shown. Images were equally adjusted for brightness using Adobe Photoshop CS5 with regard to the negative control. Magnification 10x, size bar 100 μM.

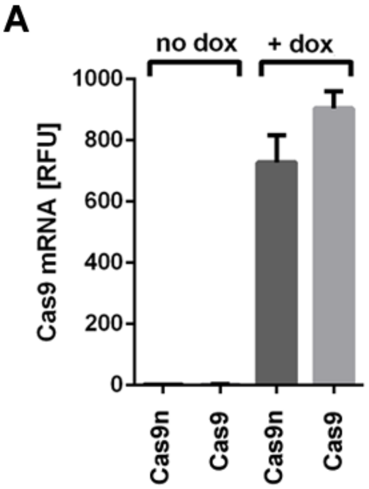

ratio of copy number (Fam/Hex)

amplicon	cell line: Cas9n	Cas9
Neo backbone	0.02	0.03
M2rtTA	0.54	0.57
Puro backbone	0.08	0.05
Cas9n/Cas9	0.50	0.46

Supplementary Fig. 8: Confirmation of inducible Cas9/Cas9n expression and absent off-target integration. Cas9 or Cas9n expression can be induced by doxycycline in 409-B2 iCRISPR cell lines (A) as shown by qPCR. Cells were treated without doxycyline or with 2µg/ml doxycycline for two days. The Cas9 mRNA level is inferred from qPCR of cDNA as relative fluorescence units (RFU). Values were normalized to *GAPDH* levels. Error bars show the standard deviation of two technical replicates each. The iCRISPR cassettes are heterozygously introduced in the AAVS1 locus and off-target integration of the iCRISPR cassettes or the plasmid backbone is absent (B) as shown by ddPCR. Presented values of the copy number ratio (Fam-insert/Hex-genomic control) are means of three technical replicates. PCR primer and dye probe sequences are shown in Supplementary Table 3.

Supplementary Table 1: Inconsistencies between studies using small molecules to increase CRISPR Precise Genome Editing (PGE). PGE percentages with an asterisk have been estimated from figures. Non significant precise genome editing changes are labelled with ns.

Targeted protein	Small molecule	Concentration [µM]	Cell type	Locus/Gene	Donor	PGE increase [%]	PGE increase [fold]	Reference
Ligase IV	**SCR7**	1	A549	*TSG101*	dsODN	n.a. (gel)	1.8	Maruyama et al. 2015[4]
		1	MelJuSo	*TSG101*	dsODN	n.a. (gel)	19.1	Maruyama et al. 2015[4]
		1000	mouse embryos	*Kell*	ssODN	28.6 to 58.3	2	Maruyama et al. 2015[4]
		1000	mouse embryos	*Igkc*	ssODN	5 to 22.7	4.5	Maruyama et al. 2015[4]
		1	HEK293A	*LMNA*	dsODN	10 to 11.5*	1.2	Pinder et al. 2015[5]
		1	HEK293/TRL	AAVS1	dsODN	5 to 25	5	Chu et al. 2015[6]
		50	mouse embryos	*Tex15*	ssODN	5.8 to 56.2	10	Singh et al. 2015[7]
		80	rabbit embryos	*RLL*	dsODN	ns		Song et al. 2015[8]
		1	hPSCs	GFP	dsODN	ns		Yang et al. 2015[9]
		200	human pre-B Rhe	pGG49	dsODN	ns (NHEJ decrease)		Greco et al. 2016[10]
		1	hiPSCs	*CTNNB1*	dsODN	ns		Zhang et al. 2017[11]
		1	hiPSCs	*PRDM14*	dsODN	ns		Zhang et al. 2017[11]
DNA-PK	**NU7026**	30	HEK293	GFP	dsODN	3 to 7.6	2.5	Suzuki et al. 2016[12]
	**NU7441**	2	MEFs	*TP53*	ssODN	3 to 10*	10	Robert et al. 2015[13]
		2	HEK293/TRL	GFP	dsODN	1.9 to 3.8	2	Robert et al. 2015[13]

		2	hiPSCs	*CTNNB1*	dsODN	13 to 16*	1.2	Zhang et al. 2017[11]
		2	hiPSCs	*PRDM14*	dsODN	ns		Zhang et al. 2017[11]
RAD51	**RS-1**	7,5	rabbit embryos	*RLL*	dsODN	4.4 to 26.1	5.9	Song et al. 2015[8]
		7,5	rabbit embryos	*CFTR*	dsODN	12.5 to 30	2.4	Song et al. 2015[8]
		10	HEK293A	*LMNA*	dsODN	3.5 to 21*	6	Pinder et al. 2015[5]
		10	U2OS	*LMNA*	dsODN	1.9 to 2.4*	1.3	Pinder et al. 2015[5]
		1	PFF	*APP*	ssODN	ns		Wang et al. 2016[14]
		10	hiPSCs	*CTNNB1*	dsODN	ns		Zhang et al. 2017[11]
		10	hiPSCs	*PRDM14*	dsODN	ns		Zhang et al. 2017[11]
β3-adrenergic receptor ?	**L755507**	5	mouse ESCs	*Nanog*	dsODN	17.7 to 33.3	2	Yu et al. 2015[15]
		5	hiPSCs	*SOD1*	ssODN	0.35 to 3.13	9	Yu et al. 2015[15]
		5	HeLa	*ACTA2*	dsODN	1 to 2*	2	Yu et al. 2015[15]
		5	HEK293A	*LMNA*	dsODN	ns		Pinder et al. 2015[5]
		5	hiPSCs	*CTNNB1*	dsODN	ns		Zhang et al. 2017[11]
		5	hiPSCs	*PRDM14*	dsODN	ns		Zhang et al. 2017[11]

Supplementary Table 2: Overview of the small molecules evaluated in this study. Literature references with an asterisk indicate the small molecule as a CRISPR-Cas effector. Abbreviations: alternative NHEJ (Alt-NHEJ), damage dependent signaling (DDS).

Pathway	Protein targeted	Protein function	Small molecule	Small molecule function	Reference
**NHEJ**	**Ku70/80**	First proteins to bind to DNA ends	**STL127685**	4-fluorophenyl analog of a Ku70/80 inhibitor	Weterings et al. 2016[16]
	**DNA-PK**	Complex of Ku70/80 and DNA-PKcs, DNA-PKcs phosphorylates itself and downstream effectors at the repair site	**NU7026**	DNA-PK inhibitor	Suzuki et al. 2016*[12]
	**Ligase IV**	End-processing ligation	**SCR7**	Ligase IV inhibitor	Maruyama et al. 2015*[4]
**Alt-NHEJ (NHEJ)**	**WRN helicase**	DNA unwinding	**NSC 19630**	WRN helicase inhibitor	Aggarwal et al. 2011[17]
**HDR**	**CtIP**	DNA end resection	**MLN4924**	NAE inhibitor, inhibts neddylation of CtIP	Jimeno et al. 2015[18]
	**RPA**	Coating and stabilization of ssDNA	**NSC15520**	Inhibits binding of RPA to p53 and RAD9	Glanzer et al. 2011[19], 2013[20]
	**RAD52**	ssDNA annealing	**AICAR**	RAD52 inhibitor	Sullivan et al. 2016[21]

	**RAD51**	Strand invasion with the donor DNA	**RS-1**	RAD51 enhancer	Song et al. 2016*[8]
			**B02**	RAD51 inhibitor	Huang et al. 2011[22]
**DDS**	**ATM**	Phosphorylates many DNA repair proteins	**Resveratrol**	Direct stimulatory effects on purified ATM	Lee et al. 2014[23]
			**Trichostatin A**	Histone deacetlyase inhibitor, induces phosphorylation of Ser1981 in ATM	Lee 2007[24]
**?**	**β3-adrenergic receptor**	Involved in activation of adenylate cyclase	**L755507**	β3-adrenergic receptor agonist	Yu et al. 2015*[15]

Supplementary Table 3: Oligonucleotides used in this study. gRNA (crRNA target) and single stranded DNA donors (ssODNs) for editing of *CALD1*, *KATNA1*, *SLITRK1*, *HPRT*, *DNMT1,* and AAVS1(BFP) as well as primers for analysis and Q5 site-directed-mutagenesis of the Cas9 iCRISPR donor plasmid are shown. Mutations are in bold letters and ancestral mutations are underlined as well.

**gRNAs**	*CALD1* t1	TGGAGACTATTGCTGCTTGA
	CALD1 t2	GCAGTATACCAGTGCAATTG
	KATNA1 t1	AAATGATGACCCTTCCAAAA
	KATNA1 t2	CAACACCTAAAATAAGGGTA
	SLITRK1 t1	GCTAACAGTTTACCCTGCCC
	SLITRK1 t1	ACCCGTCGCTATCGCTGCTG
	HPRT t1	GGTTAAAGATGGTTAAATGAT
	DNMT1 t1	CTGATGGTCCATGTCTGTTAC
	iCRISPR BFP insertion t1	TGTCGGCTGCTGGGACTCCG
	iCRISPR BFP insertion t2	TACAGCATCGGCCTGGCTAT
**ssODNs**	*CALD1* Cas9	GTATACTGCTCCAGTCTGCTGTCAATCTTGGAGACTA<u>**C**</u>TGCTGCTTGATGGGTCGATTTGACACCACTGCTAAAAAAGTAAACACATACA
	CALD1 Cas9n	TTATATGTATGTGTTTACTTTTTTAGCAGTGGTGTCAAATCGACCCATCAAGC**T**GCA<u>**G**</u>TAGTCTCCAAGATTGACAGCAGACTGGAGCAGTATACCAGTGC**T**ATTGAGGTGAGAATTGTCCTCAGCGTTATGGTCCTGCTGAACAGAAATAGA
	KATNA1 Cas9	GAAGGGTCATCATTTTCAGAAG<u>**C**</u>ACCTCCAACACCTAAAATAAGGG**A**AAGGGGAGAGTGAAAAAGATATTAAGTTGGATTATACCAAATG
	KATNA1 Cas9n	CTCATCTATATCCCAGGGAAAATTAGTAGCTGCCAGAACCATAACCATTTT**A**GAAGGGTCATCATTTTCAGA**G**G<u>**C**</u>**G**CCTCCAACACCTAAAATAA**C**GGTAAGGGGAGAGTGAAAAAGATATTAAGTTGGATTATACCAAATGAAGCT
	SLITRK1 Cas9	TGTTAGCTAAGGGTTTGTTCCTGG<u>**C**</u>GCTACCCGTCGCTATCGCTGC**G**GTGGGTCTGATTTTGATCTGCCAGTTGCCTGGGATCTTTGTAC
	SLITRK1 Cas9n	TCATCTTTAAACCCGACCCTGGGATGTGGTCGCAGCTGCAGCCCCCAGG**A**CAGGGTAAACTGTTAGCTAAGGGTTTGTTCCTGG<u>**C**</u>**A**CT**G**CCCGTCGCTATCGCTGC**G**GTGGGTCTGATTTTGATCTGCCAGTTGCCTGGGATCTTTGTACCTCCG
	HPRT Cpf1	GCCATTTCACATAAAACTCTTTTAGGTTA**T**AGATGGTTAAATGA**A**TGACAAAAAAAGTAATTCACTTACAGTCTGGCTTATATCCAACAC
	DNMT1 Cpf1	TTAACATCAGTACGTTAATGTTTCCTGAT**C**GTCCATGTCTGTTA**G**TCGCCTGTCAAGTGGCGTGACACCGGGCGTGTTCCCCAGAGTGAC

	mtagBFP-iCRISPR-Cas9n	AAAGACGATGACGATAAGATGGCCCCAAAGAAGAAGCGGAAGGTCGGTATCCACGGAGTCC<br>CAGCAGCC**GTGAGCAAGGGCGAGGAGCTGATCAAGGAGAACATGCACATGAAGCTGTACAT**<br>**GGAGGGCACCGTGGACAACCACCACTTCAAGTGCACCAGCGAGGGCGAGGGCAAGCCCTAC**<br>**GAGGGCACCCAGACCATGCGCATCAAGGTGGTGGAGGGCGGCCCCCTGCCCTTCGCCTTCG**<br>**ACATCCTGGCCACCAGCTTCCTGTACGGCAGCAAGACCTTCATCAACCACACCCAGGGCAT**<br>**CCCCGACTTCTTCAAGCAGAGCTTCCCCGAGGGCTTCACCTGGGAGCGCGTGACCACCTAC**<br>**GAGGACGGCGGCGTGCTGACCGCCACCCAGGACACCAGCCTGCAGGACGGCTGCCTGATCT**<br>**ACAACGTGAAGATCCGCGGCGTGAACTTCACCAGTAATGGGCCTGTGATGCAGAAGAAGAC**<br>**TCTGGGCTGGGAGGCATTCACCGAGACCCTCTATCCGGCTGATGGTGGGCTCGAGGGTCGC**<br>**AACGATATGGCTTTGAAACTCGTCGGAGGAAGTCACCTCATCGCAAACGCTAAAACAACCT**<br>**ATAGGTCTAAGAAGCCCGCCAAGAACTTGAAAATGCCAGGGGTCTACTATGTAGATTACCG**<br>**CTTGGAACGAATTAAAGAGGCTAATAATGAGACTTACGTAGAACAACACGAGGTAGCAGTC**<br>**GCTCGATATTGCGACTTGCCGAGTAAGCTCGGACATAAGCTGAACGGCAGTGGAGAAGGTC**<br>**GGGGATCACTCCTGACGTGTGGAGATGTTGAAGAGAACCCCGGCCCC**GACAAGAAGTACAG<br>CATCGGCCTGGCCATCGGCACCAACTCTGTGGGCTGGGCCGTGATCACCGACGAGTACAAG<br>GTGCCCA
**Primers**	*CALD1* forward	ACACTCTTTCCCTACACGACGCTCTTCCGATCTGCTAATCAGCTAGCATATGTATGAGAA
	CALD1 reverse	GTGACTGGAGTTCAGACGTGTGCTCTTCCGATCTTTGGACTTGATTATTGTCCTAAGTG
	KATNA1 forward	ACACTCTTTCCCTACACGACGCTCTTCCGATCTCCTGACGGCAAAGGAATATAG
	KATNA1 reverse	GTGACTGGAGTTCAGACGTGTGCTCTTCCGATCTACTGTGCTTCCTTGTATTGTTGT
	SLITRK1 forward	ACACTCTTTCCCTACACGACGCTCTTCCGATCTGGGCTTCAAATCAGCCAAG
	SLITRK1 reverse	GTGACTGGAGTTCAGACGTGTGCTCTTCCGATCTTTTCAAGACAAATGGGCAAG
	HPRT forward	ACACTCTTTCCCTACACGACGCTCTTCCGATCTGGTGAAAAGGACCCCACGAA
	HPRT reverse	GTGACTGGAGTTCAGACGTGTGCTCTTCCGATCTTGGCAAATGTGCCTCTCTACAAAT
	DNMT1 forward	ACACTCTTTCCCTACACGACGCTCTTCCGATCTTGAACGTTCCCTTAGCACTCTG
	DNMT1 reverse	GTGACTGGAGTTCAGACGTGTGCTCTTCCGATCTCCTTAGCAGCTTCCTCCTCC
	Q5 D10A forward	TGGTGCCGAT**AG**CCAGGCCGATG
	Q5 D10A reverse	ACTCTGTGGGCTGGGCCG
	qPCR Cas9 forward	CCGAAGAGGTCGTGAAGAAG
	qPCR Cas9 reverse	GCCTTATCCAGTTCGCTCAG
	qPCR GAPDH forward	GGAGCCAAACGGGTCATCATCTC
	qPCR GAPDH reverse	GAGGGGCCATCCACAGTCTTCT
	ddPCR control forward	AATCTACTCCCAGGAGCAG
	ddPCR control reverse	GTCTGTTTGAGGTTGCTAGTG
	ddPCR control probe	[HEX]TCAGGGCAGAGCCATCTATTGCT[BHQ1]
	ddPCR Cas9 insert forward	CTGAACGCCAAGCTGATTAC
	ddPCR Cas9 insert reverse	TTTCCACCAGCTGTCTCTT
	ddPCR Cas9 insert probe	[6FAM]TTCGACAATCTGACCAAGGCCGAG[BHQ1]
	ddPCR Puro backb forward	GGGTTACATCGAACTGGATCTC
	ddPCR Puro backb reverse	CGGCGTCAATACGGGATAAT
	ddPCR Puro backb probe	[6FAM]TAAAGTTCTGCTATGTGGCGCGGT[BHQ1]
	ddPCR M2rtTA insert forward	GCATAGAATCGGTGGTAGGT
	ddPCR M2rtTA insert reverse	TACACTGGGCTGCGTATT
	ddPCR M2rtTA insert probe	[6FAM]TTGCTACTTGATGCTCCTGTTCCTCC[BHQ1]
	ddPCR Neo backb forward	GCGCCTTATCCGGTAACTAT
	ddPCR Neo backb reverse	ACATACCTCGCTCTGCTAATC
	ddPCR Neo backb probe	[6FAM]AAGACACGACTTATCGCCACTGGC[BHQ1]

Supplementary Table 4: Effects of small molecules on Targeted Nucleotide Substitution (TNS) efficiency in *CALD1*, *KATNA1* and *SLITRK1* with Cas9n and Cas9. Shown are the cleavage enzyme, small molecules, loci, the mean absolute percentages of TNS and indels of all technical replicates from n independent experiments, and the mean fold change of TNS when using the respective small molecule compared to the control (as also shown in Fig. 2). Concentrations used were 20μM NU7026, 0.01μM Trichostatin A, 0.5μM MLN4924, 1μM NSC 19630, 5μM NSC 15520, 20μM AICAR, 1μM RS-1, 1μM Resveratrol, 1μM SCR7, 5μM L755507, 5μM STL127685 and 20μM B02.

			**Control**		**Small molecule**			
**Cleavage enzyme**	**Small molecule**	**Locus**	**Indel [%]**	**TNS [%]**	**Indel [%]**	**TNS [%]**	**n**	**mean fold change of TNS**
iCRISPR Cas9n	NU7026	*CALD1*	40,7	8,1	34,6	11,7	4	1,5
		KATNA1	63,5	8,5	49,7	18,7	4	2,6
		SLITRK1	81,7	5,2	68,7	11,9	3	2,5
	Trichostatin A	*CALD1*	34,2	6,8	31,7	10,1	5	1,5
		KATNA1	63,5	8,5	53,4	17,6	4	2,2
		SLITRK1	81,7	5,2	77,3	8,7	3	1,8
	MNL4924	*CALD1*	34,0	6,8	32,4	7,7	5	1,2
		KATNA1	63,5	8,5	63,5	8,5	4	1,1
		SLITRK1	81,7	5,2	76,2	6,6	3	1,3
	NSC 19630	*CALD1*	47,6	9,1	47,6	9,4	3	1,1
		KATNA1	63,5	8,5	60,2	8,7	4	1,0
		SLITRK1	81,7	5,2	79,4	5,5	3	1,0
	NSC 15520	*CALD1*	34,2	6,8	36,7	7,5	5	1,4
		KATNA1	63,5	8,5	62,9	8,1	4	1,0
		SLITRK1	81,7	5,2	82,2	4,8	3	1,0
	AICAR	*CALD1*	34,2	6,8	36,8	6,9	5	1,1
		KATNA1	63,5	8,5	63,4	8,0	4	1,1
		SLITRK1	81,7	5,2	81,0	5,2	3	1,0
	RS-1	*CALD1*	47,6	9,2	48,6	8,5	3	1,1
		KATNA1	63,5	8,5	62,0	8,0	4	1,2
		SLITRK1	81,7	5,2	79,0	5,3	3	1,0
	Resveratrol	*CALD1*	35,8	7,1	37,3	7,1	3	1,1
		KATNA1	62,2	7,0	63,6	8,0	3	1,0
		SLITRK1	75,8	6,4	76,0	6,2	2	1,1
	SCR7	*CALD1*	35,8	7,1	35,6	7,4	3	1,1
		KATNA1	57,9	6,3	59,1	6,3	2	1,0
		SLITRK1	80,0	5,5	77,2	5,9	2	1,0
	L755507	*CALD1*	28,8	5,8	29,3	5,5	4	1,0
		KATNA1	57,9	6,3	55,2	5,6	2	1,0
		SLITRK1	80,0	5,5	80,4	4,6	2	0,9
	STL127685	*CALD1*	43,6	8,4	42,0	7,4	2	0,9
		KATNA1	62,2	7,0	59,8	6,4	3	1,1
		SLITRK1	80,0	5,5	78,8	4,9	2	0,9
	B02	*CALD1*	32,1	10,7	29,3	6,5	1	0,6
		KATNA1	35,4	8,3	37,7	3,9	1	0,5
		SLITRK1	70,6	7,7	71,1	4,6	1	0,6
iCRISPR Cas9	NU7026	*CALD1*	31,2	15,9	18,2	25,5	2	1,5
		KATNA1	30,5	3,2	18,6	5,2	2	1,6
		SLITRK1	18,7	4,9	8,3	6,2	2	1,2
	Trichostatin A	*CALD1*	31,2	13,1	26,3	12,3	2	0,8
		KATNA1	30,5	3,2	27,8	3,4	2	1,0
		SLITRK1	18,7	4,9	15,5	5,0	2	1,0
	MNL4924	*CALD1*	31,2	13,1	25,5	10,5	2	0,7
		KATNA1	30,5	3,2	32,8	2,7	2	0,8
		SLITRK1	18,7	4,9	18,5	4,1	2	0,8
	NSC 19630	*CALD1*	45,8	21,2	44,8	21,2	1	1,0
		KATNA1	30,5	3,2	31,0	2,8	2	0,9
		SLITRK1	18,7	4,9	18,0	5,0	2	1,0
	NSC 15520	*CALD1*	31,2	13,1	31,2	15,7	2	1,3
		KATNA1	30,5	3,2	31,7	3,3	2	1,1
		SLITRK1	18,7	4,9	18,9	5,4	2	1,1
	AICAR	*CALD1*	31,2	13,1	31,8	13,7	2	1,1
		KATNA1	30,5	3,2	29,6	2,9	2	0,9
		SLITRK1	18,7	4,9	18,6	5,1	2	1,0
	RS-1	*CALD1*	45,8	21,2	43,9	22,2	1	1,0
		KATNA1	30,5	3,2	30,1	2,5	2	0,8
		SLITRK1	18,7	4,9	17,5	4,4	2	0,9
	Resveratrol	*CALD1*	31,2	13,1	31,6	13,3	2	1,1
		KATNA1	30,5	3,2	24,7	2,7	2	0,8

	SLITRK1	18,7	4,9	17,9	4,3	2	0,9
SCR7	*CALD1*	31,2	13,1	31,2	12,5	2	0,9
	KATNA1	30,5	3,2	29,8	2,8	2	0,8
	SLITRK1	18,7	4,9	18,7	4,7	2	0,9
L755507	*CALD1*	31,2	13,1	31,4	11,6	2	0,9
	KATNA1	30,5	3,2	30,4	3,4	2	0,9
	SLITRK1	18,7	4,9	18,2	3,9	2	0,7
STL127685	*CALD1*	31,2	13,1	30,3	12,8	2	1,0
	KATNA1	30,5	3,2	29,6	2,7	2	0,8
	SLITRK1	18,7	4,9	18,8	4,9	2	1,0
B02	*CALD1*	45,8	21,2	41,5	13,1	1	0,6
	KATNA1	30,5	3,2	31,1	2,0	2	0,5
	SLITRK1	18,7	4,9	16,5	2,2	2	0,4

Supplementary data references

1. Pinello L*, et al.* Analyzing CRISPR genome-editing experiments with CRISPResso. *Nat Biotechnol* **34**, 695-697 (2016).

2. Chari R, Mali P, Moosburner M, Church GM. Unraveling CRISPR-Cas9 genome engineering parameters via a library-on-library approach. *Nat Methods* **12**, 823-826 (2015).

3. O'Brien J, Wilson I, Orton T, Pognan F. Investigation of the Alamar Blue (resazurin) fluorescent dye for the assessment of mammalian cell cytotoxicity. *Eur J Biochem* **267**, 5421-5426 (2000).

4. Maruyama T, Dougan SK, Truttmann MC, Bilate AM, Ingram JR, Ploegh HL. Increasing the efficiency of precise genome editing with CRISPR-Cas9 by inhibition of nonhomologous end joining. *Nat Biotechnol* **33**, 538-542 (2015).

5. Pinder J, Salsman J, Dellaire G. Nuclear domain 'knock-in' screen for the evaluation and identification of small molecule enhancers of CRISPR-based genome editing. *Nucleic Acids Res* **43**, 9379-9392 (2015).

6. Chu VT*, et al.* Increasing the efficiency of homology-directed repair for CRISPR-Cas9-induced precise gene editing in mammalian cells. *Nat Biotechnol* **33**, 543-548 (2015).

7. Singh P, Schimenti JC, Bolcun-Filas E. A mouse geneticist's practical guide to CRISPR applications. *Genetics* **199**, 1-15 (2015).

8. Song J, Yang D, Xu J, Zhu T, Chen YE, Zhang J. RS-1 enhances CRISPR/Cas9- and TALEN-mediated knock-in efficiency. *Nat Commun* **7**, 10548 (2016).

9. Yang D, Scavuzzo MA, Chmielowiec J, Sharp R, Bajic A, Borowiak M. Enrichment of G2/M cell cycle phase in human pluripotent stem cells enhances HDR-mediated gene repair with customizable endonucleases. *Sci Rep* **6**, 21264 (2016).

10. Greco GE, Matsumoto Y, Brooks RC, Lu Z, Lieber MR, Tomkinson AE. SCR7 is neither a selective nor a potent inhibitor of human DNA ligase IV. *DNA Repair (Amst)* **43**, 18-23 (2016).

11. Zhang JP*, et al.* Efficient precise knockin with a double cut HDR donor after CRISPR/Cas9-mediated double-stranded DNA cleavage. *Genome Biol* **18**, 35 (2017).

12. Suzuki K*, et al.* In vivo genome editing via CRISPR/Cas9 mediated homology-independent targeted integration. *Nature* **540**, 144-149 (2016).

13. Robert F, Barbeau M, Ethier S, Dostie J, Pelletier J. Pharmacological inhibition of DNA-PK stimulates Cas9-mediated genome editing. *Genome Med* **7**, 93 (2015).

14. Wang K*, et al.* Efficient Generation of Orthologous Point Mutations in Pigs via CRISPR-assisted ssODN-mediated Homology-directed Repair. *Mol Ther Nucleic Acids* **5**, e396 (2016).

15. Yu C*, et al.* Small molecules enhance CRISPR genome editing in pluripotent stem cells. *Cell Stem Cell* **16**, 142-147 (2015).

16. Weterings E*, et al.* A novel small molecule inhibitor of the DNA repair protein Ku70/80. *DNA Repair (Amst)* **43**, 98-106 (2016).

17. Aggarwal M, Sommers JA, Shoemaker RH, Brosh RM, Jr. Inhibition of helicase activity by a small molecule impairs Werner syndrome helicase (WRN) function in the cellular response to DNA damage or replication stress. *Proc Natl Acad Sci U S A* **108**, 1525-1530 (2011).

18. Jimeno S, Fernandez-Avila MJ, Cruz-Garcia A, Cepeda-Garcia C, Gomez-Cabello D, Huertas P. Neddylation inhibits CtIP-mediated resection and regulates DNA double strand break repair pathway choice. *Nucleic Acids Res* **43**, 987-999 (2015).

19. Glanzer JG, Liu S, Oakley GG. Small molecule inhibitor of the RPA70 N-terminal protein interaction domain discovered using in silico and in vitro methods. *Bioorg Med Chem* **19**, 2589-2595 (2011).

20. Glanzer JG, Carnes KA, Soto P, Liu S, Parkhurst LJ, Oakley GG. A small molecule directly inhibits the p53 transactivation domain from binding to replication protein A. *Nucleic Acids Res* **41**, 2047-2059 (2013).

21. Sullivan K*, et al.* Identification of a Small Molecule Inhibitor of RAD52 by Structure-Based Selection. *PLoS One* **11**, e0147230 (2016).

22. Huang F, Motlekar NA, Burgwin CM, Napper AD, Diamond SL, Mazin AV. Identification of specific inhibitors of human RAD51 recombinase using high-throughput screening. *ACS Chem Biol* **6**, 628-635 (2011).

23. Lee JH, Guo Z, Myler LR, Zheng S, Paull TT. Direct activation of ATM by resveratrol under oxidizing conditions. *PLoS One* **9**, e97969 (2014).

24. Lee JS. Activation of ATM-dependent DNA damage signal pathway by a histone deacetylase inhibitor, trichostatin A. *Cancer Res Treat* **39**, 125-130 (2007).
